# The Use of Extracellular Vesicles as a Promising Therapeutic Approach for Pulmonary Diseases

**DOI:** 10.1002/hsr2.70853

**Published:** 2025-06-11

**Authors:** Sirous Sadeghian Chaleshtori, Zanyar Pirkani, Massoumeh Jabbari Fakhr, Mona Mokhber

**Affiliations:** ^1^ Department of Internal Medicine, Faculty of Veterinary Medicine University of Tehran Tehran Iran; ^2^ Institute of Biomedical Research University of Tehran Tehran Iran; ^3^ Department of Tissue Engineering and Applied Cell Sciences, School of Medicine Qom University of Medical Sciences Qom Iran

**Keywords:** diagnostic, extracellular vesicles, pulmonary diseases, therapeutic

## Abstract

**Background and Aims:**

Pulmonary disorders significantly impact worldwide healthcare, highlighting the necessity for enhanced diagnostic and treatment approaches. This narrative review seeks to investigate the potential of extracellular vesicles (EVs) as an innovative therapeutic strategy for diverse pulmonary disorders.

**Methods:**

This review synthesizes existing data about the function of extracellular vesicles (EVs) in pulmonary health and pathology, emphasizing their modes of action, including the transfer of proteins, nucleic acids, and lipids for intercellular communication. It also examines the implications of EVs in targeted medication delivery, immunomodulation, and tissue regeneration.

**Results:**

EVs, including microvesicles and exosomes, have demonstrated promise in treating respiratory diseases such as chronic obstructive pulmonary disease (COPD), asthma, pulmonary fibrosis, and acute respiratory distress syndrome (ARDS). Additionally, EVs may serve as valuable biomarkers for early disease detection, prognosis, and monitoring. However, challenges remain regarding the standardization of EV isolation methods and characterization protocols to ensure safety and clinical applicability.

**Conclusion:**

In summary, extracellular vesicles hold potential for transforming the management of pulmonary diseases by providing insights into pathophysiology, enabling early diagnosis, and facilitating personalized treatment approaches. Further exploration of EV‐based therapies is necessary to fully realize their potential in improving outcomes for lung disorders.

**Abbreviations**
ADMSCs: adipose‐derived mesenchymal stem cells
ADMSCs‐EVs: adipose‐derived mesenchymal stem cells derived extracellular vesicles
ALI: acute lung injury
ARDS: acute respiratory distress syndrome
AST: aspartic aminotransferase
BALF: bronchoalveolar lavage fluid
BM‐MSCs: bone marrow mesenchymal stem cells
CK: creatine kinase
COPD: chronic obstructive pulmonary disease
CRP: C reactive protein
ECM: extracellular matrix
EVs: extracellular vesicles
hUC‐MSC: human umbilical cord mesenchymal stem cell
ILs: interleukins
ILVs: intraluminal vesicles
IPF: idiopathic pulmonary fibrosis
MDVs: mitochondrial‐derived vesicles
MIP‐2: macrophage inflammatory protein‐2
MSCs: mesenchymal stem/stromal cells
MSCs‐EVs: mesenchymal stem cells‐derived extracellular vesicles
MVBs: multivesicular bodies
NK: natural killer
PGE2: prostaglandin E2
TGF‐β: transforming growth factor beta
TLRs: toll‐like receptors
TNF‐α: tumor necrosis factor alpha

## 1 Introduction

Pulmonary diseases include numerous conditions affecting the lungs and airways, representing a significant global public health issue. The World Health Organization (WHO) reported that Chronic Obstructive Pulmonary Disease (COPD) caused around 3.2 million fatalities in 2015, making it the third leading cause of death worldwide. Comparable figures underscore the seriousness of Acute Respiratory Distress Syndrome (ARDS), with an estimated yearly incidence in the United States. between 64.2 and 78.9 per 100,000 patients, and a pooled death rate over 43%. Asthma, a common respiratory condition, impacts approximately 262 million individuals globally and results in approximately 445,000 deaths each year. Pulmonary fibrosis, a progressive and frequently lethal disorder, affects roughly five million individuals worldwide, resulting in approximately 40,000 deaths in the United States annually [1].

Despite the considerable impact of pulmonary disorders, existing therapeutic choices are often limited and predominantly concentrate on symptom management rather than preventing disease progression. Conventional treatments, including bronchodilators, corticosteroids, and oxygen therapy, may offer limited relief, but their efficacy, especially in advanced stages, is frequently inadequate. In COPD, bronchodilators and inhaled corticosteroids offer clinical relief but do not alter the fundamental disease progression. In critical instances, lung transplantation may be the sole possible alternative; however, it is limited by donor availability and involves considerable risks. Likewise, the management of ARDS predominantly depends on supportive care, including breathing and nutritional assistance for patients.

Given the limitations of current treatment options, there is an urgent need for new and effective therapies for pulmonary diseases [2]. Mesenchymal stem cells (MSCs) represent a significant therapeutic approach in the treatment of pulmonary diseases, as their inherent multipotency allows cells to contribute to regeneration after lung tissue injuries [3, 4]. Preclinical studies indicate that in models of COPD and pulmonary fibrosis, MSC transplantation has been associated with a reduction in inflammatory cytokine levels by approximately 40%–50% and a decrease in collagen deposition by 30%–40% compared to controls [5, 6]. In ARDS models, cell transplantation has demonstrated a reduction in pulmonary edema by up to 35%, improved arterial blood gas parameters, and accelerated repair of lung tissue damage [7, 8].

**Figure 1:**
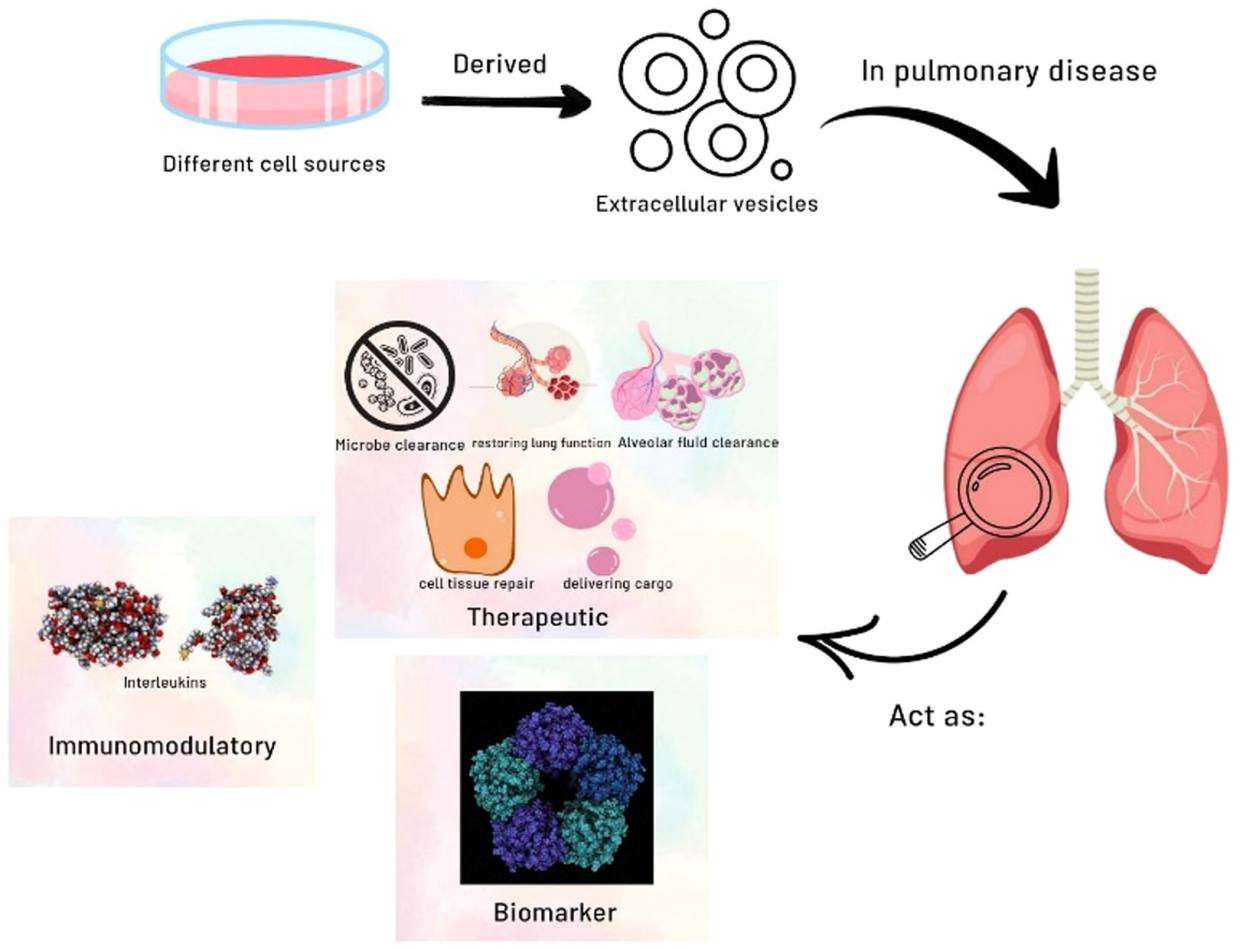
Derivation of EVs from different cell sources. EVs are a therapeutic approach for pulmonary diseases, which have this potential based on tissue repair and regeneration capabilities, immune modulation, anti‐inflammatory effects, and biomarker‐carrying capacity.

Extracellular vesicles contain a diverse cargo of proteins, nucleic acids, and lipids, making them promising therapeutic agents for various diseases, including pulmonary disorders [9, 10, 11, 12, 13]. They have this potential based on their immune modulation, anti‐inflammatory effects, tissue repair and regeneration capabilities, and their capacity to transport and serve as biomarkers [14, 15] (Figure 1). Furthermore, EVs have the capacity to be used as drug‐delivery vehicles for the management of pulmonary diseases. The studies suggest that EVs may hold promise as a novel treatment method for a broad range of respiratory disorders [16, 17].

An overview of the current state of research on EVs in the management of lung ailments is provided in this review. This article will cover EV classifications, modes of action, and their potential as pharmaceutical transport vehicles and therapeutic agents for lung disorders, including their immunomodulatory properties [18, 19, 20, 21]. Additionally, it will discuss the challenges and prospects of employing EV‐based therapies for respiratory illnesses, aiming to enhance understanding of the potential role of EVs and encourage further research in this field.

This review begins with a description of respiratory diseases and the limitations of current therapeutic techniques. We then discuss extracellular vesicles (EVs), emphasizing their formation, classification, and key characteristics. Subsequently, we explore the immunomodulatory, anti‐inflammatory, and tissue repair processes of EVs, along with their role in targeted drug delivery, highlighting their therapeutic potential in pulmonary diseases. Subsequently, we examine significant preclinical research and recent clinical trials, with particular emphasis on different routes of administration and dose considerations. Finally, we address current challenges and future goals for EV‐based therapies, including the optimization of clinical protocols and the standardization of separation techniques.

## 2 Classification and Characteristics of Extracellular Vesicles

EVs can be classified into three main types based on their biogenesis, size, and composition: exosomes, microvesicles, and apoptotic bodies [22, 23, 24].

• Exosomes (30–150 nm) are generated within endosomal compartments before their release;

• Microvesicles (100–1000 nm) originate from direct budding of the plasma membrane;

• Apoptotic bodies (50–5000 nm) are formed during the process of cell death.

All three types of EVs contain a variety of biomolecules, inclusive proteins, nucleic acids, and lipids, which can mediate intercellular communication and modulate various biological processes (Table 1).

**Table 1:** Kinds of EVs categorized by size and formation process [25, 26, 27, 28].

EV type	Size (nm)	Formation process
Exosomes	30–150	After fusing endosomes into the plasma membrane, endosomes release to the ECM
Microvesicle	100–1000	Bud directly to the ECM from the plasma membrane
Apoptotic body	50–5000	Releasing during the apoptosis phenomenon

Along all these classifications, new studies found a novel sub‐group belonging to the EV family. This novel subpopulation with districted characteristics is called mitochondrial‐derived vesicles (MDVs). Several studies highlighted this subgroup; for instance, in 2022, Picca et al. provided a new insight into MDVs' cargo, such as mitochondrial DNA, and their ability to take part in intercellular communications during aging or different disease processes [22]. Moreover, research by Zhang et al. in 2021 identified other new subpopulations like supermeres, which show to have unique biophysical properties and which are important in the intercellular signaling process [23]. EVs fffare heterogeneous in size, composition, and function, and there is significant overlap between the various kinds of EVs [24, 29].

## 3 Therapeutic Potential and Mechanisms of Action

Many different types of lung cells, inclusive endothelial cells, immune cells, and epithelial cells, release EVs [30]. EVs show their therapeutic effects in two ways, (I) exogenously by delivering cargo(small molecules, proteins, RNAs) or (II) endogenously by incorporation of therapy cargo into EVs during their biogenesis [31].

EVs have been shown to play a significant role in regulating inflammation, modulating immune responses, and repairing damaged tissue in lung diseases [32, 33, 34]. Besides their role in inflammation, EVs can also regulate the activity of neighboring cells in the lung; however, understanding the precise modulatory actions of EVs in the lungs is still an active area of research [35, 36].

### 3.1 Anti‐Inflammatory and Immunomodulatory Effects

By affecting immune cell activity or directly interacting with lung cell surface receptors like TLRs, EVs have been demonstrated to have immunomodulatory properties. They also initiate downstream signaling pathways that can control immune cell activity and modify the immune response in the lung [37, 38, 39, 40], which can be beneficial in the treating lung disorders. This may be particularly useful in illnesses like COPD and asthma, where chronic inflammation in the lung is a key feature [41, 42]. Inflammation is an intricate process that involves the activation of various immune cells, including dendritic cells and macrophages, which release pro‐inflammatory cytokines and chemokines. EVs are involved in regulating this process, both promoting and inhibiting inflammation [43, 44, 45]. For more clarity, T cell‐derived EVs can contain microRNA, which could inhibit the manufacture process of pro‐inflammatory cytokines and suppress the inflammatory response [46, 47].

EVs have been found to carry cytokines, and it was found that immune‐derived EVs play a key role in how the immune system works [30, 48, 49]. ILs are a group of cytokines, which have a vital role in regulating the immune reaction in pulmonary diseases. EVs have the ability to alter the expression and release of ILs, potentially contributing to their immune‐modulating effects in lung diseases. For example, IL‐10 is carried by regulatory T cell EVs. Interleukin‐10 is an anti‐inflammatory cytokine that can slow down the immune reaction by stopping the production of pro‐inflammatory cytokines like TNF‐α and IL‐6, lowering the recruitment of inflammatory cells, and accelerate the healing process. EVs that come from dendritic cells might carry IL‐2, a cytokine that is very important for making T cells and stimulating them. Regulatory T cell differentiation is promoted by IL‐2, which can suppress the immune response and reduce inflammation [50, 51, 52, 53].

EVs have the potential to reduce inflammation in conditions such as allergies and asthma. Scientists have discovered that EVs made from regulatory T cells in people with asthma stop the production of cytokines that cause inflammation, such as IL‐4, IL‐5, and IL‐13. These EVs enhance the production of IL‐10, thereby suppressing pro‐inflammatory cytokines for example IL‐6 and IL‐8. They have also been found to decrease the production of IL‐1β, a pro‐inflammatory cytokine that contributes to tissue damage and inflammation [54].

Overall, these findings suggest that EVs have potential as therapeutic agents in the management of pulmonary diseases through their immunomodulatory effects. The ability of EVs to transfer bioactive molecules and modulate the immune response makes them attractive candidates for the development of novel therapeutics for pulmonary diseases.

### 3.2 Promotion of Tissue Repair and Regeneration

When it comes to lung diseases, EVs can play a significant role in the regeneration and repair of damaged tissue. Neighboring cells can internalize EVs secreted by epithelial cells, which helps in tissue regeneration and repair. EVs derived from epithelial cells may contain epithelial growth factor, which may promote proliferation, as well as anti‐inflammatory chemicals that aid in reducing inflammation, which is essential for enabling the healing process to begin [55, 56, 57]. Similarly, EVs released by endothelial cells can influence the activity of neighboring cells and stimulate angiogenesis, which is a crucial process for tissue repair; They can achieve this by delivering proangiogenic growth factors [58, 59, 60, 61, 62]. EVs have shown antifibrotic properties by modulating fibroblasts activation, reducing the deposition of collagen, and promoting the regeneration of the lung tissue.

In experimental models of pulmonary diseases, MSC‐EVs have displayed a pronounced ability to decrease inflammation, promote tissue repair, and restore the function of the lungs. The human amnion epithelial cell‐EVs have been revealed to have antifibrotic, immunomodulatory, and regenerative properties in pulmonary fibrosis, which shows that EVs may have therapeutic roles in this disease as well [63, 64].

Extracellular vesicles derived from stem cells or regenerative cells may play a significant role in the regeneration and repair of lung tissue in the context of pulmonary disease [65, 66, 67]. These types of EVs carry various cargos, including growth factors, genetic materials, and cytokines, which may stimulate cellular proliferation. Additionally, they may influence angiogenesis and tissue remodeling. EVs contribute by providing regenerative signals, thereby participating in the repair process and facilitating functional recovery in lung disease [68, 69].

In ARDS, MSCs and MSC‐derived EVs have demonstrated ability to reduce fibrosis and promote lung regeneration. The therapeutic promise has been especially pertinent during the COVID‐19 pandemic, since numerous patients experienced severe ARDS. Although effective antiviral therapies have been established to treat COVID‐19, MSC‐EVs have been explored as a supplementary treatment for alleviating the inflammatory aspect of the disease. Preliminary research indicates that MSC‐EVs might reduce inflammation, enhance pulmonary function, and accelerate recovery in COVID‐19 patients [70, 71]. Furthermore, other studies have reviewed the role of EVs in the viral pulmonary infection COVID‐19 [72, 73].

### 3.3 Targeted Cargo Delivery

EVs can be designed to target specific cells and tissues in the lung, which is different from traditional drug delivery systems. This can make the therapeutic agent more effective while also reducing its side effects [31, 74, 75, 76]. EVs also have the capability to support therapeutic agents from degradation and immunological clearance, which can at least lead to an improvement in their pharmacokinetics and pharmacodynamics [77, 78, 79]. Additionally, EVs derived from specific cell kinds can be engineered to render specific cargo to targeted cells or parts of the lungs [80, 81].

One of the essential roles in which EVs can be useful in pulmonary disease treatment is delivering specific therapeutic cargo, such as siRNA, proteins, drugs, or gene‐editing tools, to target specific cells in the lungs. Researchers could program and modify EVs to investigate special surface markers or load them with therapeutic cargo [82, 83]. These modified EVs can be administered systemically or locally to the target site [84, 85]. This targeted delivery could be more helpful and accurate and also have the ability to improve the effectiveness of the therapeutic agent and decrease side effects and prescribed doses.

## 4 Preclinical Studies

There are several studies on using types of EVs in the treatment of different pulmonary diseases in animal models. An overview of the process of inducing the disease model, the type of EVs and the method of administration and the results of the studies are mentioned below (Table 2). By consolidating findings across different models, we can better understand the consistent outcomes and mechanisms through which EVs exert their effects.

**Table 2:** The different studies on using types of EVs in the treatment of pulmonary diseases model in animals. An overview of the process of inducing the disease model and the type, dose and the administration method of EVs.

Animal model	Condition or disease	Source	Dose	Rout of administration	Reference
Mouse	Sepsis‐induced lung injury	ADMSCs‐EVs		—	[86]
Horse	Natural severe asthma	PBN‐EVs		Endobronchial	[87]
Mouse	Mechanical ventilation ‐induced lung injury	EC‐EXs	300 µg EVs Single dose	Intratracheal	[88]
Mouse	Ovalbumin‐ induced asthma	ADMSCs‐EVs	10 μg EVs Four dose in four days	Intranasal	[89]
Rat	Cigarette smoke‐induced COPD	hUCMSC‐EVs	EVs isolated from 2.5 × 10^6^ hUC‐MSCs Single dose	Intratracheal	[90]
Mouse	LPS‐induced ALI	BMMSCs‐EXs		Intratracheal	[91]
Rat	*E. coli*‐induced ALI	hUCMSC‐EVs	EVs isolated from 3.5‐4 × 10^7^ hUC‐MSCs (100 million EVs/kg) Single dose	Intravenous	[92]
Mouse	Bleomycin‐induced PF	hBMMSCs‐EVs	EVs isolated from 5 × 106 hBM‐MSCs (8.6 × 10^8^ particles) Single dose	Intravenous	[93]
Mouse	Ischemia/reperfusion‐induced lung injury	BMMSCs‐EXs	EVs isolated from 2 × 10^6^ hBM‐MSCs Single dose	Intratracheal	[94]
Mouse	Ovalbumin‐ induced asthma	hADMSCs‐EVs	37 μg EVs Single dose	Intravenous	[95]
Mouse	*Aspergillus* hyphal extract‐induced AAI	hBMMSCs‐EVs	EVs isolated from 3 × 10^6^ hBM‐MSCs Single dose	Intravenous	[96]
Mouse	*E. coli* pneumonia ‐induced ALI	hBMMSCs‐MVs	MVs isolated from 1 × 10^6^ hBM‐MSCs Single dose	Intratracheal	[97]
Mouse	*E. coli* endotoxin‐induced ALI	hBMMSCs‐MVs	MVs isolated from 3 × 10^6^ hBM‐MSCs Single dose	Intratracheal	[98]

Abbreviations: ADMSCs‐EVs, adipose derived mesenchymal stem cells‐derived EVs; ALI, acute lung injury; AAI, allergic airway inflammation; BMMSCs‐EXs, bone marrow mesenchymal stem cells‐derived exosomes; EC‐EXs, epithelial cells‐derived exosomes; EVs, extracellular vesicles; hUCMSCs‐EVs, human umbilical cord mesenchymal stem cells‐derived EVs; LPS, lipopolysaccharide; EVs; PF, pulmonary fibrosis. MVs, microvesicles; PBN‐EVs, peripheral blood neutrophils‐derived.

### 4.1 Acute Lung Injury (ALI) and ARDS

Recent studies have shown that EVs play a crucial role in the pathophysiology of ALI and ARDS. Shen et al. demonstrated that EVs derived from adipose‐derived mesenchymal stem cells (ADMSCs) effectively reduced airway inflammation in a sepsis model of lung injury in mice. In a mice model of lung injury caused by sepsis and damage to alveolar epithelial cells caused by lipopolysaccharide, exosomes from ADMSCs were found to be very important for activating autophagy. The delivery of circular RNA Fryl (circ‐Fryl) and the regulation of the miR‐490‐3p/SIRT3 path specifically achieved this [86].

In another study by Sui et al. in 2021, in the mice model, ALI was induced via intraperitoneal administration of lipopolysaccharide, and delivered exosomes derived from BM‐MSCs intratracheally. Administering exosomal lncRNA‐p21 suppressed epithelial cell apoptosis and mitigated lung damage, potentially by increasing sirtuin‐1 (SIRT1) expression and regulating the miR‐181/SIRT1 pathway. Their results indicated that exosomes have novel therapeutic potential for the treatment of ALI [91].

Varkouhi et al. used *E. coli* to create an ALI model in rats and EVs from human MSCs taken from the umbilical cord to be injected intravenously. Their study has demonstrated the enhanced survival of these EVs and their ability to control ALI. EVs were observed to have a reduction effect on the alveolar‐arterial oxygen gradient, along with a decrease in alveolar protein leakage. Additionally, the level of alveolar TNF‐α had diminished. On the other hand, there was an increase in lung mononuclear phagocytes, as well as an elevation in endothelial nitric oxide synthase within the damaged lung [92, 99].

Wang et al. conducted another study in 2022; they used epithelial cell‐derived exosomes intratracheally to induce lung injury in mice using mechanical ventilation. Their investigation resulted in demonstrating that exosomes released by epithelial cells have the ability to be internalized via macrophages. This caused macrophage M2 polarization. Exosomes derived from epithelial cells have been found to comprise miR‐21a‐5p, as demonstrated. These exosomes promote macrophage M2 polarization through downregulation of the Notch2/SOCS1 signaling pathway. Recipients undergoing mechanical ventilation experience such downregulation in their macrophages [88].

In 2015, Monsel et al. studied the therapeutic effects of human bone marrow MSC‐derived microvesicles in a mouse model of severe pneumonia ALI with *E. coli*, which was injected into the trachea. Their study revealed that the microvesicles could enhance bacterial clearance, reduce the bacterial load, reduce lung injury, inflammation, and permeability of lung proteins, and increase the level of monocyte phagocytosis, thereby promoting lung tissue repair and improving survival [97].

Zhu et al. in 2014 worked on *E. coli* endotoxin intratraceally‐induced ALI in a mouse model. They used microvesicles extracted from human BM‐MSC, intratracheally. Their research showed that using those microvesicles could greatly lower the amounts of total protein in bronchoalveolar lavage fluid (BALF) and extravascular lung water. This means that less protein could pass through the lungs and there was less swelling in the lungs. Similarly, microvesicles declined the influx of inflammatory cells and alveolar MIP‐2 in BALF. They stated that microvesicles can reduce airway inflammation as well as improve the function of the lungs [98].

The transmission of antiapoptotic miR‐21‐5p as cause of protection provided by BMMSCs‐derived exosomes in a mouse model of lung ischemia/reperfusion damage was showcased in the study conducted by Li et al. [94]. They caused damage to the lungs by cutting the thorax and blocking the hilar artery in the left lung. The lungs were then re‐perfused and exosomes from BM‐MSCs were delivered through the trachea. The analysis conducted has established a noteworthy decrease in pulmonary edema and impairment, the alveolar macrophages polarization to the M1 phenotype, and the secretion of inflammatory cytokines. Oxidative stress induced a significant decrease in apoptosis, and exosomal miR‐21‐5p targeted both PDCD4 and PTEN in the lung.

### 4.2 Asthma

In asthma, EVs released by airway smooth muscle cells contribute to airway remodeling. Research conducted by Mainguy‐Seers et al. in 2022 indicated that EVs released by peripheral blood neutrophils may significantly influence the proliferation and growth of airway smooth muscle cells in a natural model of severe asthma in horses. EVs contain matrix proteins and growth factors that promote the differentiation and proliferation of airway smooth muscle cells, contributing to airway remodeling development [87].

In a 2021 study by Mun et al., mice with asthma induced by ovalbumin injection received intranasal delivery of EVs from ADMSCs. This treatment led to a significant decline in allergic airway inflammation and mitigation of airway hyperresponsiveness, characteristic features of asthma [89]. The EVs derived from ADMSCs, have shown a strong inhibitory effect on the overall amount of eosinophils and inflammatory cells present in the BALF. This has a big impact on improving the lung pathology that is usually linked to this condition [89].

De Castro et al. in 2017 found that EVs could significantly reduce the counts of eosinophils, IL‐4, and IL‐5 in mouse lung tissue, as well as the counts of eosinophils and total leukocyte in BALF. Moreover, they observed a reduction in the counts of CD3 + CD4 + T cells in both the lung and the thymus. ADMSCs‐EVs also decreased the amount of collagen fibers in the lung parenchyma and airways, as well as the level of TGF‐β in the lung tissue. This changed how the lungs were remodeled [95].

In another study by Cruz et al. in 2015, it was conducted that systematic administration of EVs, which are extracted from human BM‐MSCs, could have a role in repressing Th2/Th17‐interceded airway hyperresponsiveness as well as inflammation in the lung in the model of allergic airway inflammation caused by receiving *Aspergillus* hyphal extract through the oropharynx in mice [96, 100].

### 4.3 Chronic Obstructive Pulmonary Disease (COPD)

In 2021, Ridzuan et al. investigated the therapeutic potential of EVs derived from human umbilical cord mesenchymal stem cell (hUC‐MSC) in a cigarette smoke‐induced COPD model in rats. Their study showed that putting hUC‐MSC‐released EVs into the trachea could lower inflammation, thickening of the alveolar septum, and the number of goblet cells, as well as improve lung function in an animal model of COPD. Additionally, their findings showed that hUC‐MSC‐derived EVs significantly modulate various pathways associated with COPD [90].

Chen et al. contributed to the existing literature by showing that hUC‐MSCs reduce inflammation in the lungs of mice modeled by acute cigarette smoke extract‐induced pulmonary inflammation. In addition, hUC‐MSCs lessen lung apoptosis after injection. In the short‐term CS‐exposed pulmonary inflammation paradigm, they found that hUC‐MSCs can lower inflammation and apoptosis. One potential new approach to treating acute pulmonary inflammatory disease is hUC‐MSCs [101].

Genschmer et al. investigated the function of neutrophil‐derived exosomes in the development of COPD. It was found that these exosomes contain elastase and are increased in the plasma of people with COPD. Administration of these exosomes to mice induced emphysema, indicating a possible therapeutic target for COPD treatment [102].

### 4.4 Pulmonary Fibrosis (PF)

There is a significant unmet medical need for effective pulmonary fibrosis therapies. Basalova et al. assessed the efficacy of components of the secretome of mesenchymal stromal cells (MSCs) in preventing the development of pulmonary fibrosis and aiding in its resolution. Mice injured by bleomycin instillation had their lung fibrosis prevented by intratracheal administration of MSC‐EVs or MSC‐SF, a vesicle‐depleted secretome fraction, but this was unexpected. The vesicle‐depleted fraction failed to resolve existing pulmonary fibrosis, while MSC‐EV treatment caused it to resolve. Myofibroblast and FAPa+ progenitor counts were reduced following MSC‐EV administration; however, their apoptotic potential was unaffected. This decline was probably brought about by the dedifferentiation they underwent as a result of the miR transfer induced by MSC‐EVs. The antifibrotic activity of MSC‐EVs was validated by using a rodent model of bleomycin‐induced lung fibrosis, wherein we identified miR‐29c and miR‐129 as specific miRNAs. By utilizing the vesicle‐enriched fraction of the MSC secretome, their discovery offers fresh perspectives on potential antifibrotic treatment [103].

A study by Mansouri et al. investigated the effects of intravenous administration of EVs derived from human BM‐MSCs in mice with bleomycin‐induced pulmonary fibrosis. EVs modulated lung macrophage phenotypes. The bone marrow displayed an immunomodulatory effect. Giving EV therapy led to the growth of a type of monocyte that controls the immune system and reduces inflammation. This treatment also improved the main symptoms of bleomycin‐induced pulmonary fibrosis and inflammation in the lung [93].

These studies collectively suggest that EVs hold promise as a novel treatment method for various pulmonary diseases. However, optimizing their use as therapeutic agents in this context is essential for maximizing their effectiveness.

## 5 Clinical Trials

Clinical trials examining EVs for pulmonary conditions remain in preliminary phases but exhibit encouraging potential. The majority of research has been on ARDS and pulmonary disorders associated with COVID‐19, predominantly using EVs derived from bone marrow mesenchymal stem cells (BM‐MSCs). A summary of registered clinical trials accessible on“https://clinicaltrials.gov/ct2/home”in which EVs have been used to treat lung disease are given in Table 3. A finalized phase II trial (NCT04493242) evaluated the efficacy and safety of ExoFlo, an extracellular vesicle product generated from bone marrow mesenchymal stem cells, for moderate to severe ARDS associated with COVID‐19. The research indicated that the 15 mL dosage of ExoFlo was safe for patients with severe or critical respiratory failure related to COVID‐19.

**Table 3:** List of some of clinical trials working on using EVs in pulmonary diseases treatment accessible on (https://clinicaltrials.gov/ct2/home).

Clinical trial ID	Status	Condition or disease	study	Phase
NCT05354141	Recruiting	ARDS	To evaluate the efficacy and safety of intravenous delivery of BM‐MSCs derived EVs, ExoFlo, versus placebo	Phase III
NCT05061212	Not yet recruiting	ARDS	The Mechanism of EVs comprising mitochondrial DNA in ARDS lung damage induced by extrapulmonary sepsis	—
NCT04493242	Completed	COVID‐19 ARDS	To estimate the efficacy and safety of intravenous delivery of BM‐MSCs derived EVs, ExoFlo, versus placebo	Phase II
NCT05787288	Recruiting	COVID‐19 Pneumonia	Investigate the efficacy and safety of nebulized inhalation of EVs derivative from MSCs combined with standard treatment for COVID‐19 patients	Phase I
NCT03857841	Terminated	Bronchopulmonary dysplasia	A study the safety of intravenous infusion of BM‐MSCs derivative EVs, (UNEX‐42) in premature neonates at high danger for bronchopulmonary dysplasia	Phase I
NCT05127122	Not Yet Recruiting	ARDS	Evaluation of the efficacy and safety of intravenous delivery of BM‐MSCs derivative EVs, ExoFlo versus saline	Phase I & II
NCT05116761	Not Yet Recruiting	Post‐Acute COVID‐19 and Chronic Post‐COVID‐19 syndrome	Assessment of the efficacy and safety of intravenous delivery of BM‐MSCs derivative EVs, ExoFlo	Phase I & II
NCT05125562	Withdrawn	mild‐moderate COVID‐19	Evaluation of the efficacy and safety of intravenous delivery of BM‐MSCs derivative EVs,	Phase II
NCT04657458	available	COVID‐19 assosiated ARDS	BM‐MSCs derived EVs, infusion treatment	—
NCT05808400	Recruiting	Chronic cough after COVID‐19	Estimation of the efficacy and safety of UC‐MSC derivative EVs nebulization inhalation therapy for therapy of chronic cough after COVID‐19 infection	Phase I

A notable Phase I clinical trial (NCT04276987) was registered in China to investigate MSC‐EVs as a therapy for ARDS secondary to COVID‐19. While full results are pending publication, this study aimed to evaluate the efficacy and safety of EV treatment in severe cases.

The Phase II trial NCT04602104 is assessing the efficacy and safety of exosomes generated from mesenchymal stem cells for severe COVID‐19 pneumonia. The trial NCT04491240 is examining the application of MSC‐derived exosomes for moderate‐to‐severe ARDS. A phase III multicenter, randomized, double‐blinded, placebo‐controlled trial (NCT05354141) is presently assessing the efficacy and safety of ExoFlo for the management of hospitalized patients with moderate‐to‐severe ARDS, based on these findings.

These trials are essential for gathering data on optimal dosing regimens, effective administration routes, and therapeutic outcomes. The urgency created by the COVID‐19 pandemic has accelerated research into EV applications for lung disorders, potentially offering new therapeutic alternatives for various pulmonary conditions.

As the research advances, extensive, meticulously designed clinical studies with standardized protocols will be essential to ascertain the efficacy and safety profiles of EV treatments in lung disorders, fulfilling the requirement for multi‐targeted therapy in complicated circumstances such as severe COVID‐19 and ARDS. Research has shown that EV treatments can effectively modulate inflammatory responses and promote tissue regeneration following lung injuries caused by various factors, including viral infections like SARS‐CoV‐2. Studies indicate that EV treatments can reduce inflammation, support alveolar epithelium restoration, regeneration microvascular permeability, and prevent fibrosis in lung injury models.

## 6 Administration Routes and Dosing Considerations

The therapeutic effectiveness of EVs in pulmonary and systemic disorders is reliant not only upon their cargo and origin but also significantly on their method of delivery. Recent research have investigated several administration routes—such as intravenous, intratracheal, intranasal, and nebulization—to enhance tissue targeting, biodistribution, and clinical results.

### 6.1 Administration Routes

#### 6.1.1 Intratracheal and Intranasal Delivery

Localized delivery techniques, like intratracheal instillation and intranasal treatment, are especially advantageous for pulmonary ailments as they facilitate elevated local EV concentrations while minimizing systemic exposure. Chen et al. illustrated that intratracheal use of human umbilical cord mesenchymal stem cell (hUC‐MSC)–derived EVs in the rat model of cigarette smoke‐induced pulmonary inflammation markedly reduced inflammatory markers and enhanced lung histopathology relative to systemic injection [104]. Likewise, intranasal methods have been evaluated in preclinical models of neurological illnesses (e.g., intranasal EV delivery enhanced motor symptoms in rodent models), underscoring the possibility for targeting central nervous system tissues via this noninvasive technique.

#### 6.1.2 Intravenous Delivery

Intravenous (IV) infusion is the principal method employed in clinical trials. Studies indicate that intravenous delivery leads to significant accumulation of extracellular vesicles in off‐target organs, chiefly the liver and spleen, which could reduce their therapeutic concentration in target tissues like the lung or heart. Tolomeo et al. the biodistribution of human MSC‐derived extracellular vesicles in mice after intravenous, intratracheal, and intranasal injection. Biodistribution was evaluated at 3 and 24 h following administration using whole‐body imaging and organ analysis. Results demonstrated that intravenous treatment resulted in EV formation predominantly in the liver and spleen, indicating systemic distribution. IT administration led to EV localization predominantly in the lungs, signifying successful pulmonary targeting. In administration demonstrated considerable extracellular vesicle presence in the brain, indicating potential for central nervous system targeting. The research determined that the method of administration markedly affects MSC‐EV biodistribution, with intratracheal injection being more advantageous for pulmonary treatments [105]. Investigations in large animal models, particularly nonhuman primates (NHPs), provide valuable insights that both corroborate and refine the understanding gained from rodent studies. Nonhuman primates' studies in pig‐tailed macaques confirm the rapid liver and spleen accumulation of intravenous EVs observed in rodents, reinforcing the role of the reticuloendothelial system in their clearance. However, a key difference is the significantly longer circulatory persistence of EVs in macaques, suggesting rodent models may underestimate EV exposure in primates. This pharmacokinetic distinction has important implications for designing effective EV‐based therapies for clinical translation [106].

#### 6.1.3 Nebulization/Inhalation Therapy

Recent clinical studies have increasingly concentrated on nebulization as a noninvasive method for delivering EVs directly to the respiratory system. Nebulized EV formulations have been assessed regarding COVID‐19. Shi et al. [107] and later studies by Zhu et al. [108] indicated that aerosolized inhalation of adipose‐derived and umbilical cord‐derived MSC‐EVs was safe for both healthy volunteers and COVID‐19 patients, demonstrating improvements in oxygenation parameters and the resolution of pulmonary lesions in the treated individuals. Furthermore, novel formulations like Zofin—an EV‐enriched product sourced from perinatal tissues—have been delivered through nebulization in compassionate‐use scenarios for severely ill COVID‐19 patients, with research demonstrating favorable tolerability and clinical enhancement [109, 110, 111].

In summary, intratracheal and intranasal delivery methods provide enhanced lung targeting and increased local concentrations of EVs, which reduces systemic exposure. In contrast, intravenous infusion, although easier to administer, frequently results in considerable off‐target accumulation in organs such as the liver and spleen, which may reduce the pulmonary therapeutic effect. Nebulization offers a noninvasive method that integrates targeted pulmonary delivery and enhances patient compliance; however, issues related to formulation stability and dose consistency persist. These approaches emphasize the necessity of choosing a delivery strategy that is compatible with the clinical context and therapeutic goals for EV‐based treatments in pulmonary diseases.

### 6.2 Dosing Considerations

Determining the ideal dosage for EV treatments is one of the most challenging difficulties facing clinical translation. In contrast with traditional small‐molecule pharmaceuticals, EVs are complicated biological entities whose efficacy is determined not only by their concentration but also by their cargo composition, purity, and the intrinsic heterogeneity of the vesicle population [112].

Numerous research have now implemented dosing techniques predicated on either particle quantity or protein concentration. However, these measurements may be misleading if evaluated independently. For example, protein‐based quantification fails to consider variations in vesicle purity or the relative amount of bioactive cargo, whereas particle number measures could vary depending on the detection thresholds of nanoparticle tracking analysis (NTA) or flow cytometry systems. Consequently, there is an increasing demand for the creation of functional potency assays that directly correspond to therapeutic outcomes. One strategy involves quantifying specific effector molecules—such as critical miRNAs or cytokines—on the surfaces of EVs or within their lumen, so creating a “potency unit” that could provide a more dependable foundation for dose standardization [113, 114]. The following two tables detail the relationship between EV size and origin and their biodistribution [106, 115, 116] (Tables 4 and 5).

**Table 4:** The effect of size on EVs biodistribution.

Feature	Small EVs (sEVs)	Medium/Large EVs (m/lEVs)
Typical size range	~30–150 nm (or < 200 nm)	~150–1000 nm (or > 200 nm), up to 5000 nm for APBs
Circulation half‐life	Variable: Often rapid (minutes) ^37^, but reports of longer half‐lives exist (hours), potentially influenced by source/surface markers (e.g., CD47) & species	Generally rapid clearance (minutes), potentially faster than sEVs due to efficient RES uptake & filtration
Primary accumulation organs (IV Admin.)	Initial (< 1–2 h): Liver (major), Spleen, Kidneys Later (> 2–12 h): Liver, Spleen (peak often later)	Initial (< 1 h): Lungs (major, transient), Liver Later (> 2–12 h)**:** Liver (increases as lung decreases), Spleen (low), Kidneys (low/moderate)
Key clearance cells/mechanisms	RES/MPS: Liver (Kupffer Cells & LSECs‐LSECs preferential for ≤ 100 nm), Spleen (Macrophages).	RES/MPS: Liver (Kupffer Cells preferential for > 150 nm), Spleen (Macrophages).

**Table 5:** The effect of source on EVs biodistribution.

EV source (Cell type/tissue)	Key surface molecules (Examples)	Primary target organs/cells (observed/proposed)	Putative targeting mechanism (ligand‐receptor if known)	Therapeutic relevance/function example	Representative snippet IDs
Cancer cells (general)	Integrins (α6β4, α6β1, αvβ5, etc.), Tetraspanins (Tspan8), EGFRvIII, Glycans	Liver, Lungs, Spleen, Bone, Brain (depends on cancer type); Stromal cells, Endothelial cells, Immune cells	Integrin‐ECM/receptor binding; Ligand‐receptor interactions	Pre‐metastatic niche formation, Angiogenesis, Immune suppression, Transfer of oncogenes	[117]
Pancreatic cancer	Integrins (αvβ5, Tspan8‐α4, CD49d, CD151‐α3), CD106	Liver (Macrophages), Pancreas (Endothelial cells), Lungs (Fibroblasts), Aortic Endothelium	Integrin αvβ5‐Macrophages; Tspan8‐α4 ‐ CD54; CD49d‐VCAM1	Liver metastasis, Angiogenesis	[117]
Breast cancer (bone‐tropic)	Integrin‐binding sialoprotein (IBSP)	Bone (Osteoclasts)	IBSP ‐ Osteoclast receptors	Bone metastasis	[118]
Immune cells (general)	MHC complexes, Costimulatory molecules, Integrins (e.g., αM on Platelet EVs), Tetraspanins (CD9, CD81)	Lymphoid organs (Spleen), Liver, Site of inflammation; Other immune cells (DCs, T cells, Monocytes)	Antigen presentation; Integrin‐receptor binding (e.g., αM‐TAM receptors); Tetraspanin interactions	Immune modulation (activation/suppression), Antigen presentation	[119]
Dendritic cells (DCs)	CD9, CD81, MFG‐E8/Lactadherin, MHC‐II	Spleen (DCs)	CD9/Integrin αvβ3/αL interactions	Antigen presentation, Immune activation (Vaccines)	[120]
Macrophages	Immune proteins	Liver (high uptake), Brain vessel endothelial cells	Specific surface components mediating BBB crossing & liver uptake	Inflammation, Immune response, Potential BBB delivery	[120]
Mesenchymal stem cells (MSCs)	Integrins, Tetraspanins, Adhesion molecules	Liver, Spleen, Lungs (general); Sites of injury/inflammation (potential); Tumor sites (potential)	General RES uptake; Potential specific interactions at injury/tumor sites	Tissue repair, Angiogenesis, Immunomodulation, Anticancer effects (variable)	[121]
Red blood cells (RBCs)	Specific surface markers (unspecified)	Liver, Bone	Unknown	Physiological roles (e.g., coagulation ‐ historically)	[122]
Platelets	Integrin αM	Monocytes, Granulocytes	Integrin αM ‐ TAM receptor binding	Hemostasis, Inflammation	[122]
Neurons/neural stem cells (NSCs)	Amyloid precursor protein (APP), Other neuronal markers	CNS (Neurons, Glia), Ischemic brain regions	APP‐neuronal receptor binding; Homing to injury	Neuroprotection, CNS drug delivery, Stroke recovery	[122]

## 7 Challenges and Future Directions

EVs offer a promising approach for treating respiratory disorders, although several issues and challenges must be addressed before widespread clinical use. EV separation and characterisation require greater standardization, which is one such issue [123]. The quantity and quality of EVs obtained may vary depending on the best method for separating them, which could ultimately impact how effective they are as a treatment [124, 125]. Another significant issue that requires consideration is the capacity of EV production. Addressing this requires developing innovative cell‐free technologies capable of generating EVs in vast quantities while preserving their therapeutic value [126].

The heterogeneity of EVs presents an additional challenge. EVs represent a diverse group characterized by various subtypes, each possessing distinct biological properties. Identifying and determining these subtypes is essential for a better understanding of their therapeutic potential and for improving clinical effectiveness [125].

Another issue that needs to be considered is the safety of using EVs. Despite their potential as regeneration agents, it is essential to conduct a thorough investigation into the safety of EVs before using them widely clinically. This involves a detailed investigation into their toxic properties, pharmacology, and geographic spread [25, 127, 128].

Despite the problems we've discussed, using EVs to treat lung diseases is more complicated than we thought. This is because we don't fully understand how they work and where they go in the body. Due to the small size and complex nature of EVs, studying their pharmacokinetics and biodistribution remains challenging. More research is needed to fully understand these aspects [129, 130].

Studies on the effectiveness of EV‐based interventions for therapy are ongoing, although further research is necessary to improve their clinical use [131, 132]. Numerous scientific studies have looked into the use of EV‐based therapies as a possible treatment for lung diseases like asthma, COPD, and IPF. The results showed that EV‐based therapies could be a useful alternative for managing these debilitating lung diseases. Nevertheless, more study is required to fully understand the mechanisms behind these effects and to create safe and effective EV‐based therapies.

## 8 Conclusion

The ongoing research and development of EV‐based therapies has the potential to revolutionize the treatment of pulmonary diseases and significantly improve patient outcomes. It is thus vital to continue exploring the potential of EV‐based treatments to maximize their therapeutic advantages. To fully understand how EVs work and improve their therapeutic effectiveness, more research is needed on the complex interactions between EVs and different types of cells in pulmonary diseases. Standardization of EV isolation and characterization methods is also needed. continued research efforts, EVs are valuable tools for improving pulmonary disease management and patient outcomes in the near future.

## Author Contributions

**Sirous Sadeghian Chaleshtori:** project administration, conceptualization, validation, data curation, supervision, writing – review and editing. **Zanyar Pirkani:** conceptualization, visualization, investigation, resources, writing – original draft. **Massoumeh Jabbari Fakhr:** validation, data curation, writing – review and editing. **Mona Mokhber:** investigation, resources, writing – original draft.

## Disclosure

All authors have read and approved the final version of the manuscript and Zanyar pirkani and Sirous Sadeghian Chaleshtori had full access to all of the data in this study and takes complete responsibility for the integrity of the data and the accuracy of the data analysis.

## Ethics Statement

The authors have nothing to report.

## Conflicts of Interest

The authors declare no conflicts of interest.

## Transparency Statement

The lead author Sirous Sadeghian Chaleshtori, Zanyar Pirkani affirms that this manuscript is an honest, accurate, and transparent account of the study being reported; that no important aspects of the study have been omitted; and that any discrepancies from the study as planned (and, if relevant, registered) have been explained.

## Data Availability

The authors have nothing to report.
